# Evaluating Artificial Intelligence and Traditional Learning Tools for Chest X‐Ray Interpretation: A Descriptive Study

**DOI:** 10.1111/tct.70139

**Published:** 2025-07-12

**Authors:** Gurtek Singh Samra, Vashisht Ramoutar, Kelley Chen, Muiz Chaudhry, Hrithika Patel, Terese Bird, Vanessa Rodwell

**Affiliations:** ^1^ Department of Medicine University of Leicester Leicester UK; ^2^ Ulverscroft Eye Unit, School of Psychology and Vision Sciences University of Leicester Leicester UK

**Keywords:** artificial intelligence, asynchronous, chest X‐ray, medical education, radiology, technology enhanced learning

## Abstract

**Background:**

Chest X‐ray (CXR) interpretation is a fundamental yet challenging skill for medical students to master. Traditional resources like Radiopaedia offer extensive content, while newer artificial intelligence (AI) tools, such as Chester, provide pattern recognition and real‐time feedback. This study aims to evaluate Radiopaedia and Chester's effectiveness as educational tools and to explore student perspectives on AI.

**Approach:**

A teaching session on CXR interpretation fundamentals was delivered to establish a standardised baseline of knowledge among participants, followed by a live tutorial introducing students to the functionality of both Chester AI and Radiopaedia. Students engaged with both tools to answer a 25‐item workbook assessing complex CXR pathologies. CXRs were deliberately selected for their complexity to examine student engagement with online learning tools amid diagnostic uncertainty, encouraging applied clinical reasoning.

**Evaluation:**

Preclinical medical students were recruited and randomly assigned to the Chester AI (*n* = 5) or Radiopaedia group (*n* = 5). During the workbook task, participants were instructed to engage with the workbook using Radiopaedia and Chester AI. Post‐session, participants took part in focus groups to share their experiences. Thematic analysis highlighted Chester's efficiency and potential as a revision tool but noted limitations with complex CXR pathologies. Radiopaedia was valued for its comprehensiveness but was less efficient for the workbook task due to its vast array of content.

**Implications:**

AI tools such as Chester show promise as complementary resources alongside traditional learning materials. Combining Chester's efficiency and real‐time feedback with Radiopaedia's in‐depth content may optimise learning and improve CXR interpretation skills.

## 1 Background

The interpretation of chest X‐rays (CXRs) is a fundamental skill for medical students and healthcare professionals. However, mastering this skill can be challenging, particularly for pre‐clinical students [1]. Traditional educational resources, such as textbooks and websites like Radiopaedia [2], are commonly used to teach CXR interpretation [3]. Radiopaedia, a free online radiology resource written by radiologists, serves as a comprehensive repository of medical knowledge [4]. While such resources are comprehensive, they can be overwhelming and difficult to navigate.

In recent years, artificial intelligence (AI) has emerged as a promising tool in CXR analysis [5], providing new opportunities for clinical practice [6], alongside radiology education [7]. While some studies have reported comparable outcomes to traditional methods [8], others have highlighted potential negative impacts on learner performance [9]. Nevertheless, a significant gap remains in the literature regarding the overall effectiveness of AI in undergraduate radiology education.

**FIGURE 1:**
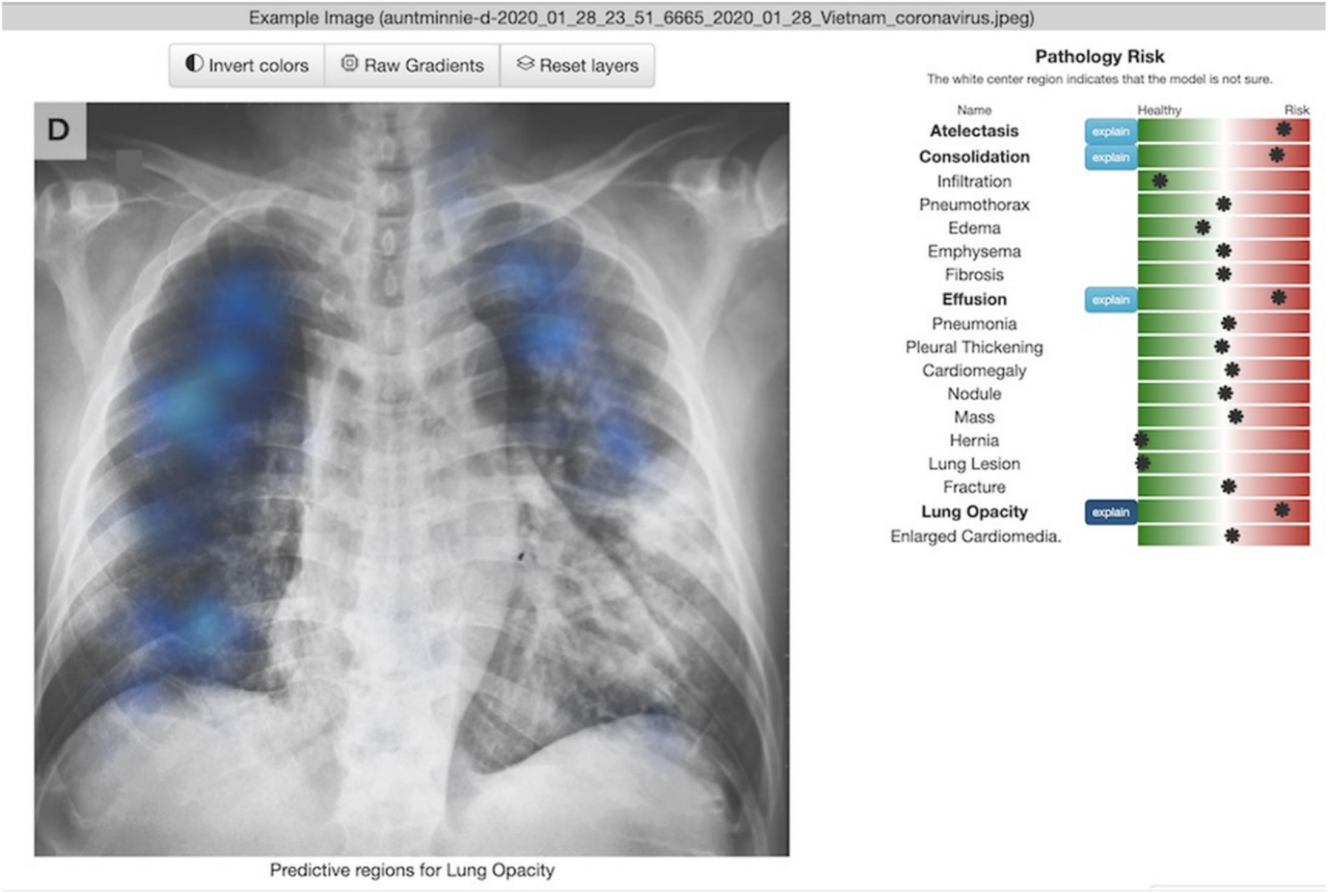
Chester AI tool, showing pathology risk scores and a saliency map for predictive Lung Opacity regions on a CXR. Image is used under fair use from open website “Chester The AI Radiology Assistant” (https://mlmed.org/tools/xray/).

*Chester the AI Radiology Assistant* (Chester AI) is a free machine‐learning browser‐based tool designed to interpret CXRs by identifying pathologies and providing probability scores for various conditions [10]. Chester AI also generates saliency maps to highlight areas of potential pathology (Figure 1).

‘*Chester the AI Radiology Assistant* (Chester AI) is a free machine‐learning browser‐based tool designed to interpret CXRs by identifying pathologies and providing probability scores for various conditions’.

In this study, we aimed to evaluate Radiopaedia and Chester AI as tools for CXR interpretation among preclinical medical students and to explore students' perceptions of both tools. Additionally, we sought to provide insights to inform tutors on the integration of AI‐based resources in radiology education.

## 2 Approach

To evaluate Chester AI and Radiopaedia as tools for CXR interpretation, a structured teaching and assessment approach was implemented. First, an interactive teaching session covering the fundamentals of CXR interpretation was delivered to establish a standardised baseline of knowledge among the preclinical medical students. This was followed by a tutorial introducing students to the functionality of both Chester AI and Radiopaedia, ensuring familiarity with the platforms before use.

The workbook (see supplement) was designed in accordance with the institution's Learning Objectives as a tool to facilitate student engagement with Radiopaedia and Chester AI. The workbook contained 25 multiple‐choice questions (MCQs), each featuring a CXR that required students to apply systematic interpretation and diagnostic reasoning. The objective was to simulate real‐life clinical scenarios where junior clinicians, when faced with difficult or ambiguous clinical interpretation tasks, may supplement their knowledge and support their clinical reasoning with diagnostic tools or clinical knowledge repositories.

CXR cases were selected to challenge students beyond their preclinical knowledge base, prompting them to seek assistance from Radiopaedia or Chester AI to aid their decision‐making process. This allowed us to examine how students interact with these online tools when encountering uncertainty, mirroring real‐world clinical situations. This task aimed to replicate that experience in an educational setting, fostering skills in information retrieval, critical appraisal and applied clinical reasoning.

## 3 Evaluation

The study was approved by the Institution's Ethics Committee (Reference [redacted for peer‐review]).

Pre‐clinical medical students from [redacted for review] were invited to participate voluntarily. Students were randomly assigned to either the Chester AI group (*n* = 5) or Radiopaedia group (*n* = 5). The study included a 25‐item MCQ workbook session, followed by focus groups. Focus groups were conducted for both groups by authors GSS and VR, aiming to gather individual perspectives while encouraging interaction among participants [11]. Transcripts were line‐by‐line coded by KC, MC and HP, with key concepts grouped into emerging themes. Assistant moderator notes were reviewed to account for group dynamics. Throughout, researchers regularly compared thematic interpretations to ensure agreement on the emerging themes.

### 3.1 Results

The median [IQR] for the Radiopaedia group was 15 [10–17] out of a possible 25 marks, while the Chester AI group had a median score of 13 [12–16]. The median time to complete the workbook was 26 min [22–26] for the Radiopaedia group and 23 min [17–24] for the Chester AI group. There were no statistically significant differences in marks or time for completion (*p* > 0.05).

Key themes, quotes and concepts identified during focus group discussions are outlined in Tables 1 and 2.

**TABLE 1:** Key themes and student perceptions of Chester AI and Radiopaedia in the workbook task.

Themes	Sub‐themes	Concepts—radiopaedia	Quotes
Educational use	Revision tool	Good revision tool	‘Good learning tool in terms of explanation underneath the [CXR]’ (Radiopaedia group)
			‘I'd use it less as a diagnostic tool, more like in the other way, like, okay, I'm going to revise pneumonia’ (Radiopaedia group)
			‘If I just wanted to check [a CXR pathology], I think radiopaedia would be great’ (Radiopaedia group)
	Reference/diagnostic tool	Good reference if you already know the diagnosis	‘If you just had the X‐ray with no history, more likely to use AI than Radiopaedia. But if I had a little bit of information, Radiopaedia is great’ (Radiopaedia)
			‘I sort of had an idea of kind of what I thought it was first, and then went to radio’ (Radiopaedia group)
	Assistive tool	Good tool to support prior knowledge	‘But if I had a little bit of information to go off, or I sort of kind of recognise it [CXR pathology], but just wanted to check, I think Radiopaedia would be great’ (Radiopaedia group)
Efficiency	Time efficiency	Manual processes take longer	‘It would just take forever to just like search through all the possible conditions’ (Radiopaedia group)
			‘Just so much information’ (Radiopaedia group)
			‘Frantically scouring Radiopaedia trying to find things’ (Radiopaedia group)
	Content	Information overload	‘Really hard just to find a simple, like, chest X‐ray of tuberculosis’ (Radiopaedia group)
			‘[Content] Not really relevant to me right now’ (Radiopaedia group)
			‘It would just take forever to just like search through all the possible conditions’ (Radiopaedia group)
			‘Just so much information’ (Radiopaedia group)
	Ease of use	Familiar tools are beneficial	‘Summary at the bottom … I found that quite useful’ (Radiopaedia group)
			‘Easier in the filter section’ (Radiopaedia group)
		Difficulty to navigate	‘Didn't find it straightforward to use’ (Radiopaedia group)

**TABLE 2:** Key themes and student perceptions on AI in CXR interpretation.

Quotes
‘Think an AI would be more accurate than me in identifying like areas of consolidation’ (Radiopaedia group)
‘If it was more accurate it would be useful in intense situations’ (Chester AI group)
‘Comes in with like a very dangerous injury … it can give the response straight away’ (Chester AI group)
‘Assesses more, perhaps, risk [of developing a disease]’ (Chester AI group)
‘AI picks up the changes and person interprets them’ (Radiopaedia group)
‘Should be, like, checked over by an actual person’ (Radiopaedia group)
‘do not think it should be used to teach’ (Chester AI group)
‘If it comes up with a similar thing to you, you can have more confidence in it; if not, I value human opinion over the AI’ (Radiopaedia group)
‘Like as a sector, you are putting someone's life at risk’ (Chester AI group)
‘Rely on it solely, is also very dangerous, like, for patient outcomes’ (Chester AI group)
‘you still need the experience of a radiologist who has lived through many different examples who has that knowledge. But I guess still human input would be needed given the anomalies that are there, for the time being’ (Chester AI group)
‘It's best to have a rudimentary understanding of how to read an x‐ray before going on to Chester to give the diagnosis for like a given image’ (Chester AI group)
‘So long as we do not over‐rely on it, in case it crashes’ (Radiopaedia group)
‘Do not want to be relying too heavily on it’ (Chester AI group)
‘If it ended up replacing radiologists’ (Chester AI group)
‘An AI tool is consistently better than the radiologist. Why would I put people's lives in the hands of somebody who's less accurate’ (Chester AI group)
‘5% chance doesn't work … you've got a whole team of experienced radiologists who can take over when it does happen’ (Chester AI group)

### 3.2 Summary of Impressions—Chester AI

Students in the Chester AI group highlighted efficiency as one of its primary advantages. Students particularly valued Chester AI's real‐time feedback, highlighting its ability to provide a visual summary of clinically relevant findings in a CXR. This efficiency was perceived as beneficial for quick‐fire quizzes and pattern recognition training, making Chester AI a useful adjunct to traditional learning materials.

However, several limitations were noted. Students found that Chester AI lacked detailed explanations, making it less useful for deeper learning. Some students reported difficulty interpreting Chester's outputs, leading to uncertainty in their conclusions. The tool was perceived as useful for obvious pathologies but less reliable for subtler or ambiguous cases, leading some students to question its dependability in clinical practice. Furthermore, the lack of reference images or additional learning materials also hindered its ability to serve as a stand‐alone educational resource.

### 3.3 Summary of Impressions—Radiopaedia

Radiopaedia was valued for its depth of information and familiarity among students. Many participants appreciated its comprehensive content, particularly for in‐depth exploration of CXR pathologies. Some students felt that prior experience with Radiopaedia made it easier to navigate, with features like filter sections and structured summaries helping them locate relevant information more efficiently.

However, within the context of the workbook task, students found Radiopaedia to be inefficient. The platform's wealth of information, while valuable for broad learning, led to ‘information overload’ when trying to answer specific questions quickly. Many students described the process of manually searching for conditions as time‐consuming, making it difficult to work through the workbook efficiently. Overall, while Radiopaedia was highly regarded for its educational value, its vast array of content limited its usefulness in time‐sensitive settings.

## 4 Implications

In this study, we compared the effectiveness of Radiopaedia and Chester AI for CXR interpretation among preclinical medical students and explored students' experiences and perceptions of both tools. Our findings highlight both the strengths and limitations of each tool, suggesting potential areas for improvement and applicability in radiology education.

A prior study by Dao et al also studied Chester AI in the context of medical education, however the study focused solely on quantitative data, and no significant differences were ellicited in diagnostic performance when comparing the AI‐assisted group to t control groups [12]. Cheng et al. developed an AI tool called *HipGuide* to assist students in detecting hip fractures on pelvic radiographs, which, similar to Chester, generates saliency maps that highlight areas of potential fractures [13]. Their study showed higher diagnostic accuracy in the AI‐assisted group compared with the control group. Our focus group findings align with this, as students perceived Chester AI as a valuable diagnostic aid that improved efficiency and reinforced pattern recognition. However, limitations in interpretability and vague outputs impacted confidence in decision‐making, suggesting that AI assistance may be most effective when combined with structured teaching.

‘Students perceived Chester AI as a valuable diagnostic aid that improved efficiency and reinforced pattern recognition’.

Our qualitative data found that Chester AI offered promise in augmenting traditional educational resources like Radiopaedia, particularly in enhancing students' confidence in image interpretation. Chester has instant feedback provided by its heatmaps and probability‐based suggestions, which make it a more engaging and interactive tool. Key themes included the tool's versatility and its potential use as a revision resource. This aligns with previous research, suggesting that accessible AI tools for asynchronous learning may be more practical and appealing than formal integration into structured teaching programmes [14]. However, our findings also suggest that the utility of these tools should be viewed as complementary rather than substitutive. Chester AI's strength lies in its pattern recognition, accessibility and time efficiency, but its limitations in dealing with complex cases and lack of detailed explanations mean that it cannot yet replace more comprehensive educational resources.

‘Chester AI's strength lies in its pattern recognition, accessibility and time efficiency, but its limitations in dealing with complex cases and lack of detailed explanations mean that it cannot yet replace more comprehensive educational resources’.

Our study findings also raise important questions about the future of AI in radiology education. Students raised concerns regarding the use of AI in clinical settings, such as the accuracy of AI models and the potential for AI to replace human radiologists. These concerns are reflective of views in the global medical community about the role of AI in radiology [15], highlighting the need for careful consideration of how such tools are integrated into both clinical and educational environments.

Our study has several limitations to note. The generalisability of the findings is constrained by several factors, including volunteer participation, a small sample size and recruitment from a single institution, which may affect the broader applicability of our study. Also, Chester AI is still in its prototype phase; therefore, the findings may not fully reflect the potential of AI tools in CXR education once they are further developed.

Furthermore, we plan to continue working on this project by repeating our study with a larger sample size to collect sufficient data to compare groups quantitatively and evaluate this alongside the qualitative data. Seeing as Chester AI is still in its early stages, it would also be interesting to see how results may differ when this becomes more developed.

In conclusion, combining Radiopaedia's comprehensive content with Chester AI's efficient pattern recognition offers a powerful approach to CXR interpretation and radiology education. While Chester AI facilitates rapid revision and real‐time feedback, Radiopaedia offers the depth needed for a more thorough understanding. To maximise learning outcomes, tutors should also promote critical evaluation of AI‐generated results, fostering clinical reasoning and diagnostic confidence.

## Author Contributions

**Gurtek Singh Samra:** investigation, methodology, writing – review and editing, writing – original draft, project administration, formal analysis, validation, data curation. **Vashisht Ramoutar:** investigation, writing – original draft, methodology. **Kelley Chen:** formal analysis, methodology, investigation, writing – original draft. **Muiz Chaudhry:** investigation, methodology, formal analysis, writing – original draft. **Hrithika Patel:** investigation, methodology, formal analysis, writing – original draft. **Terese Bird:** conceptualization, investigation, writing – review and editing, methodology, validation, visualization, supervision, resources. **Vanessa Rodwell:** conceptualization, investigation, methodology, validation, visualization, writing – review and editing, writing – original draft, supervision, resources, project administration.

## Ethics Statement

The study was approved by the Institution's General Research Ethics Committee (Ethics Reference: 0391; Project Title: Artificial Intelligence versus Traditional Chest X‐Ray Interpretation for Undergraduate Medical Students; PI: Gurtek S Samra).

## Conflicts of Interest

The authors declare no conflicts of interest.

## Supporting information


**Data S1** Supporting Information

**Data S2** Supporting Information

## Data Availability

The data that support the findings of this study are available from the corresponding author upon reasonable request.
