# Supplementary material for: Evaluating Artificial Intelligence and Traditional Learning Tools for Chest X‐Ray Interpretation: A Descriptive Study
Source: Clin Teach. 2025 Jul 12;22(4):e70139. doi: 10.1111/tct.70139 (PMC12254926; doi:10.1111/tct.70139)
Supplement: Supplementary file 1 — Data S1 Supporting Information [file TCT-22-e70139-s001.docx]

# Topic Guide

### Understanding Participants' Background

- Can you describe your familiarity and comfort level with using AI technology in the medical field, particularly for diagnostic purposes?
- What prior experience do you have with traditional methods of CXR interpretation, such as using resources like Radiopaedia?

### Exploring AI / Traditional Interpretation

- Chester: what was your initial impression of the AI tool compared to traditional methods like Radiopaedia?
- Chester/Radiopaedia: How did your experience with influence your confidence in interpreting CXRs during the experiment?
- Chester/Radiopaedia: Were there any specific features or aspects of the tool that stood out to you during the interpretation process?
- Chester/Radiopaedia: Can you discuss any challenges you encountered while using the tool? How did you navigate these challenges?

### Perceptions of Diagnostic Accuracy and Confidence

- How did your confidence levels in interpreting CXRs change from before using the assigned tool to after the interpretation session?
- What factors do you think contribute to differences in diagnostic accuracy between AI and traditional methods?
- How would you describe the learning experience when using your assigned tool?

### Preference and Future Adoption

- Chester: Based on your experience, do you have a preference for using AI-assisted diagnosis or traditional methods in your future practice? Why or why not?
- Chester: Are there any improvements or additional features you would like to see in AI tools to enhance their usability and effectiveness?

### Ethical and Social Implications

- Chester/Radiopaedia: What ethical considerations, if any, do you think are important to address when integrating AI technology into medical education and practice?
- Chester/Radiopaedia: How do you perceive the role of AI in shaping the future of diagnostic medicine, particularly among medical students and healthcare professionals?
